# Codelivery of dihydroartemisinin and chlorin e6 by copolymer nanoparticles enables boosting photodynamic therapy of breast cancer with low-power irradiation

**DOI:** 10.1093/rb/rbad048

**Published:** 2023-04-28

**Authors:** Jing Jia, Wenping Chen, Long Xu, Xuewen Wang, Min Li, Bin Wang, Xiangyu Huang, Tao Wang, Yang Chen, Mengdie Li, Dan Tian, Junyang Zhuang, Xinhua Lin, Ning Li

**Affiliations:** Fujian Key Laboratory of Drug Target Discovery and Structural and Functional Research, School of Pharmacy, Fujian Medical University, Fuzhou 350122, China; Key Laboratory of Nanomedical Technology (Education Department of Fujian Province), School of Pharmacy, Nano Medical Technology Research Institute, Fujian Medical University, Fuzhou, Fujian 350122, China; Fujian Key Laboratory of Drug Target Discovery and Structural and Functional Research, School of Pharmacy, Fujian Medical University, Fuzhou 350122, China; School of Materials Science and Chemical Engineering, Ningbo University, Ningbo 315211, China; Fujian Key Laboratory of Drug Target Discovery and Structural and Functional Research, School of Pharmacy, Fujian Medical University, Fuzhou 350122, China; Fujian Key Laboratory of Drug Target Discovery and Structural and Functional Research, School of Pharmacy, Fujian Medical University, Fuzhou 350122, China; Fujian Key Laboratory of Drug Target Discovery and Structural and Functional Research, School of Pharmacy, Fujian Medical University, Fuzhou 350122, China; Fujian Key Laboratory of Drug Target Discovery and Structural and Functional Research, School of Pharmacy, Fujian Medical University, Fuzhou 350122, China; School and Hospital of Stomatology, Fujian Stomatological Hospital, Fujian Medical University, Fuzhou 350002, China; Department of Hepatobiliary Surgery, Fuzhou Second Hospital, Fuzhou 350007, China; Fujian Key Laboratory of Drug Target Discovery and Structural and Functional Research, School of Pharmacy, Fujian Medical University, Fuzhou 350122, China; Fujian Key Laboratory of Drug Target Discovery and Structural and Functional Research, School of Pharmacy, Fujian Medical University, Fuzhou 350122, China; Fujian Key Laboratory of Drug Target Discovery and Structural and Functional Research, School of Pharmacy, Fujian Medical University, Fuzhou 350122, China; Key Laboratory of Nanomedical Technology (Education Department of Fujian Province), School of Pharmacy, Nano Medical Technology Research Institute, Fujian Medical University, Fuzhou, Fujian 350122, China; Fujian Key Laboratory of Drug Target Discovery and Structural and Functional Research, School of Pharmacy, Fujian Medical University, Fuzhou 350122, China

**Keywords:** dihydroartemisinin, photodynamic therapy, combination therapy, nano drug delivery systems

## Abstract

Given that chemotherapy as a stand-alone therapeutic strategy may not be sufficient to effectively treat cancer, there is increasing interest in combination of chemotherapy and alternative therapies. Photodynamic therapy has the advantages of high selectivity and low side effects, so the combination of photodynamic therapy and chemotherapy has become one of the most appealing strategies for tumor treatment. In this work, we constructed a nano drug codelivery system (PPDC) to realize the combined treatment of chemotherapy and photodynamic therapy through encapsulating chemotherapeutic drug dihydroartemisinin and photosensitizer chlorin e6 in PEG-PCL. The potentials, particle size and morphology of nanoparticles were characterized by dynamic light scattering and transmission electron microscopy. We also investigated the reactive oxygen species (ROS) generation and drug release ability. The antitumor effect *in vitro* was investigated by methylthiazolyldiphenyl-tetrazolium bromide assays and cell apoptosis experiments, and the potential cell death mechanisms were explored by ROS detection and Western blot analysis. The *in vivo* antitumor effect of PPDC was evaluated under the guidance of fluorescence imaging. Our work provides a potential antitumor treatment approach and expands the application of dihydroartemisinin for breast cancer therapy.

## Graphical Abstract

**Figure:**
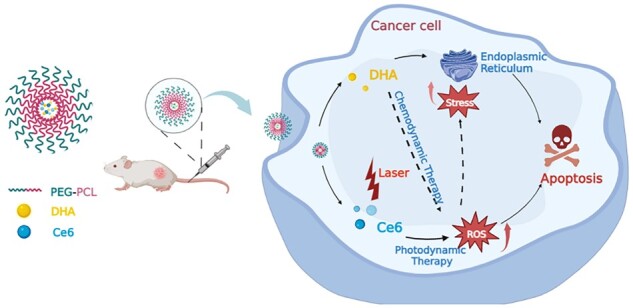

## Introduction

Cancer—one of the leading causes of death—is regarded as a major life-threatening disease worldwide. Medical researchers and scientists devote themselves to developing safe, effective and innovative medicines and therapies for cancer therapy. To date, among various therapies, chemotherapy remains an important treatment modality for tumor therapy. However, conventional chemotherapy suffers from serious side effects and multidrug resistance. Thus, it is still a challenge to develop new drugs that are safe and highly efficient. Indeed, drug discovery and development is a time-consuming and costly process [1]. Therefore, new applications of old drugs have received increasing attention. Some traditional Chinese medicines such as artemisinin (ART)—which is the first-line antimalarial drug isolated by Tu’s group—have been found to possess the antitumor potential. Dihydroartemisinin (DHA) is a derivative and the primary active metabolite of the natural compound ART; it has been applied worldwide by World Health Organization (WHO) as an effective antimalarial drug and proven to be safe with low side effects. Recent studies have shown that DHA may serve as an anticancer chemotherapeutic drug. However, the application of free DHA is limited by its instability, rapid clearance from blood circulation, and especially poor solubility. Therefore, developing an effective vehicle for DHA is urgently needed.

Recently, the development of nanoparticles and nanomedicines has received extensive attention due to their good biocompatibility and functional flexibility [2–6], which can improve the solubility of drugs, achieve passive tumor targeting via enhanced permeability and retention (EPR) effects, and ultimately improve the therapeutic efficacy of drugs [7–9]. Among various carriers, PEG-PCL copolymer is one of the widely used drug delivery vehicles because of its amphiphilic nature, good biodegradability and biocompatibility [10, 11].

In addition to the focus on the new uses for old drugs, there is increasing interest in the development of emerging novel therapeutic approaches such as photodynamic therapy (PDT) [12, 13], photothermal therapy [14, 15] and immunotherapy [16]. As a noninvasive therapy, PDT can improve the intracellular reactive oxygen species (ROS) level through exogenous regulation to kill cancer cells [17], which is an effective and promising treatment method in cancer therapy. Excessive ROS can damage lipids, proteins and DNA, thereby leading to cell death. When ROS generation increases or clearance decreases, the cell enters an abnormal growth state of oxidative stress [18]. Studies on the development of cancer have found that tumor cells are more susceptible to ROS damage induced by exogenous factors due to abnormal growth and high level of oxidative stress compared with normal cells [19, 20]. Therefore, increasing ROS levels through exogenous regulation is a method of selectively killing tumor cells and reducing the toxicity to normal cells [17, 19]. PDT induces cancer cell damage by making photosensitizer molecules react with oxygen molecules under the laser irradiation with a specific wavelength in local tumor tissues to generate singlet oxygen (^1^O_2_), which is selective to the treatment site and has slight side effects on normal tissue. Chlorin e6 (Ce6) is a commonly used photosensitizer in PDT and can produce a large amount of ^1^O_2_ under 660 nm laser irradiation and has the advantage of strong tissue permeability. However, the clinical application of Ce6 is limited for its poor solubility in water, easy agglomeration *in vivo*, low accumulation in tumor tissue and easy elimination. Therefore, it is highly desirable to develop nanotechnology-based drug delivery systems for PDT.

**Scheme 1.**
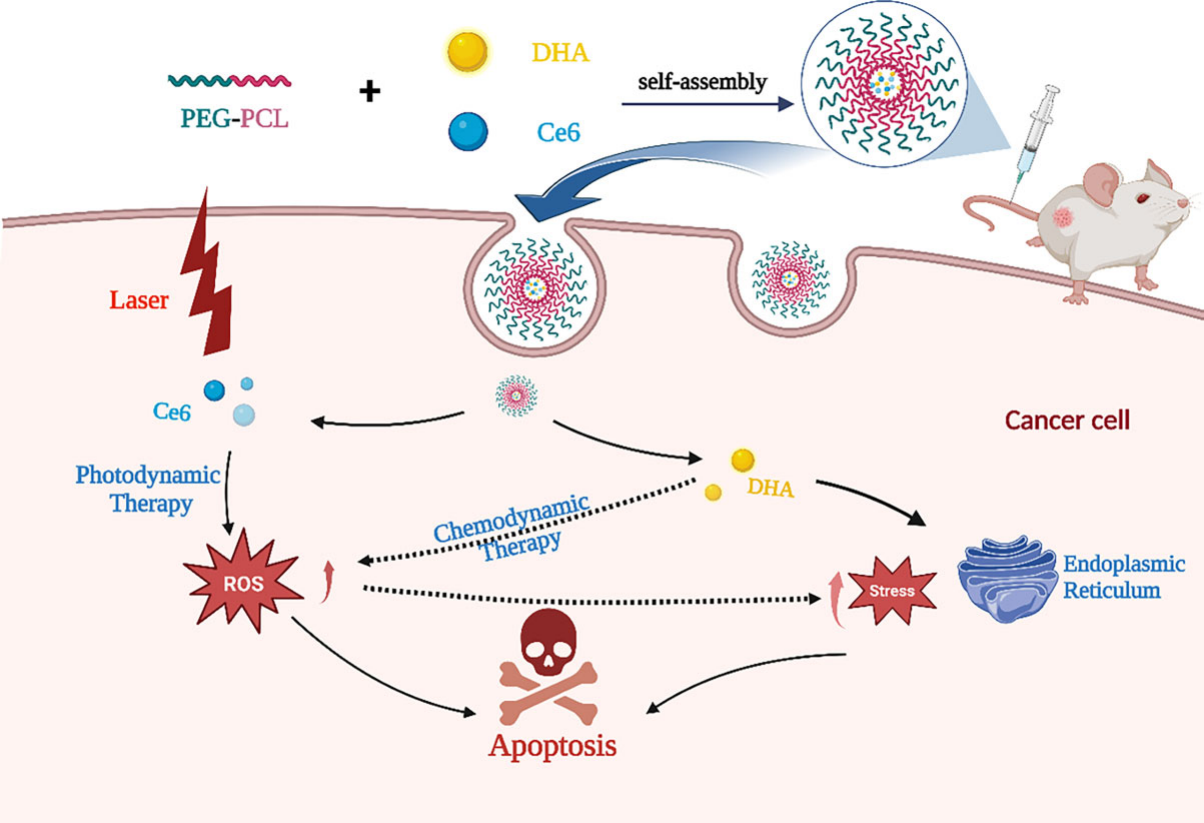
The schematic diagram of DHA&Ce6@PEG-PCL (PPDC) nanoparticles mediated codelivery of chemotherapy drug DHA (dihydroartemisinin) and photosensitizer Ce6 (chlorin e6).

Based on the above, to combine the advantages of PDT and chemotherapy, here we constructed a nano drug codelivery system (PPDC) for the combined treatment of chemotherapy and PDT. As shown in Scheme 1, PPDC with high drug loading was prepared via encapsulating the chemotherapeutic drug DHA and photosensitizer Ce6 in PEG-PCL. After PPDC was injected into 4T1 tumor-bearing mice, nanoparticles were enriched at the tumor site through the EPR effect, and the characteristic of tumor acidic environment caused the degradation of nanoparticles and drug release. DHA inhibited the growth of the tumor through a variety of injury mechanisms. PDT was activated by laser irradiation at the tumor site to achieve the combined treatment of chemotherapy and PDT. Both of them jointly improved ROS level in different ways and finally achieved the purpose of inhibiting the tumor growth by inducing endoplasmic reticulum (ER) stress and enhancing cell apoptosis. This work provides potential strategies for expanding the application of DHA and for the tumors treatment.

## Materials and methods

### Materials

DHA was purchased from Shanghai Aladdin Biochemical Technology Co., Ltd. (Shanghai, China). Ce6 was obtained from Frontier Scientific Chemicals (Logan, UT, USA). 1,3-Diphenylisobenzofuran (DPBF) was purchased from Sigma-Aldrich (St. Louis, MO, USA).

Roswell Park Memorial Institute (RPMI)-1640, Dulbecco’s modified Eagle’s medium (DMEM) and penicillin/streptomycin were bought from Adamas (Shanghai, China). Hoechst 33342 stain solution was obtained from Beijing Solarbio Science & Technology Co., Ltd (Beijing, China). Hexamethylene amiloride, Chlorpromazine HCl (CPZ) and BCA protein assay kit were purchased from Glpbio (CA, USA). Nystatin was bought from Yeasen Biotechnology Co., Ltd (Shanghai, China). Triton X-100, SDS-PAGE Gel Preparation Kit, 2-(4-Amidinophenyl)-6-indolecarbamidine dihydrochloride (DAPI), Methylthiazolyldiphenyl-tetrazolium bromide (MTT), RIPA lysis buffer, HRP-labeled Goat Anti-Mouse IgG (H + L), HRP-labeled Goat Anti-Rabbit IgG (H + L), ECL Chemiluminescence Kit and ROS Assay Kit were bought from Beyotime Biotechnology (Shanghai, China). Annexin V-FITC Apoptosis Detection Kit was purchased from Dojindo Laboratories (Japan). Bax antibody, C/EBP (CCAAT enhancer binding protein)-homologous protein (CHOP) mouse monoclonal antibody and β-actin rabbit monoclonal antibody were obtained from Cell Signaling Technology, Inc. (CST, Boston, USA).

In this study, a laser (Changchun Laser Optoelectronics Technology Co., Ltd Changchun, China) with 660 nm was selected for irradiation.

### Preparation of nanoparticles

We prepared DHA&Ce6@PEG-PCL (PPDC) by a dialysis method. In brief, PEG-PCL (12 mg), Ce6 (4 mg) and DHA (4 mg) were dissolved in 900 µl dimethyl sulfoxide (DMSO). Then, the mixture was slowly added dropwise into rapidly stirring pure water. The solution was stirred overnight and dialyzed in deionized water for 10 h to remove unloaded drugs and DMSO. Finally, after freeze-drying, we obtained PPDC (dark green solid powder).

### Characterization of nanoparticles

To further characterize the successfully prepared PPDC nanoparticles, the ultraviolet absorption spectrum and fluorescence emission spectrum were detected by an ultraviolet–visible spectrophotometer (Shimadzu, UV-2600, Japan) and a fluorescence spectrophotometer (Agilent Technologies, USA), respectively. The content of DHA and Ce6 loaded in PPDC was measured by ultraviolet absorption spectrophotometry. First, free DHA and Ce6 were prepared into solutions with different concentrations. DHA was dissolved in a mixed solvent of water containing 2% sodium hydroxide and absolute ethanol (water/ethanol = 4:1, V/V) and the absorbance at 238 nm was detected after heating at 60°C for 30 min. Ce6 was dissolved in DMSO and the absorbance at 664 nm was detected. Then, according to the mentioned method, the content of DHA and Ce6 loaded in PPDC was detected and calculated. Meanwhile, the drug loading efficiency (LE) and the encapsulation efficiency (EE) of DHA and Ce6 in PPDC were obtained via the following formulas: where *m*_1_ and *m*_2_ represent the measured mass and initial mass of drugs (DHA or Ce6) in PPDC, respectively.


(1) $$LE\;(\%)=\frac{m_{1}}{m(\text{PPDC})}\times 100\%$$

(2) $$\text{EE}\;(\%)=\frac{m_{1}}{m_{2}}\times 100\%,$$

The particle size and zeta potentials of PEG-PCL (0.5 mg/ml) and PPDC (0.5 mg/ml) were measured with a dynamic light scattering (DLS) particle size analyzer (Anton Paar, Austria). The morphologies of PEG-PCL (5 mg/ml) and PPDC (5 mg/ml) were imaged by transmission electron microscopy (TEM, Tecnai G2, FEI Company, USA) using the phosphotungstic acid negative staining method.

### ROS generation detection *in vitro*

DPBF is widely used as a ^1^O_2_ probe because it has high sensitivity to ^1^O_2_ and has a low cost to be commercially available [21]. When combined with ^1^O_2_, DPBF forms endoperoxide and decomposes into 1,2-dibenzoylbenzene (DBB), which is irreversible, leading to the decrease in the fluorescence emission intensity and ultraviolet absorption intensity of DPBF [22]. Therefore, the ^1^O_2_ generation can be judged via monitoring the attenuation of fluorescence emission (400–600 nm) and ultraviolet absorbance (417 nm). It is worth noting that in consideration of the outstanding ROS generation performance of Ce6, a low power density laser (5 mW/cm^2^) was used for the measurements.

Ce6 and PPDC solution (equivalent to Ce6 concentration: 0.63 μg/ml) were mixed with DPBF (10 μg/ml). After laser irradiation (5 mW/cm^2^) for different periods of time, the fluorescence emission and ultraviolet absorbance intensity of DPBF were detected. Meanwhile, pure DPBF solution was set as the control. Moreover, the dependence of the ROS generation efficiency on PPDC concentration or laser power was further investigated. Specifically, the mixed solution of DPBF (10 μg/ml) and PPDC with different concentrations (including 0.08, 0.16 and 0.32 μg/ml Ce6) was irradiated for 3 min, or the mixed solution (10 μg/ml DPBF and 0.32 μg/ml PPDC) was irradiated with laser of different powers (0.005, 0.05 and 0.1 W/cm^2^), and then the fluorescence emission spectra of DPBF were detected.

### Drug release assays

Drug release of PPDC was evaluated by the dialysis method. To simulate the release of DHA and Ce6 from PPDC in different physiological environments, 4.7 mg PPDC was dissolved in 160 μl phosphate-buffered saline (PBS; pH 5.0 or 7.4). Then they were added in dialysis bags (molecular weight cut-off = 3.5 kD), which were immersed in a tube containing 1.5 ml PBS with corresponding pH value. At the fixed time points (0.5, 1, 1.5, 2, 2.5, 3, 4, 6, 8, 12 and 24 h), 250 μl of the PBS was taken out accompanied by an equal volume of fresh PBS replenished. The drug concentration (DHA and Ce6) was obtained via detecting the absorption value at the wavelengths of 238 and 664 nm, respectively. The cumulative release ratio was calculated according to the following equation through three parallel experiments [23]: in which *E* represents the cumulative release amount (%), *V_E_* represents the sampling volume (250 μl), *V*_0_ represents the initial volume (1.5 ml), *C_i_* and *C_n_* represent the measured drug concentrations (μg/ml), *i* and *n* represent the sampling times and *m*_0_ represents the quality of DHA or Ce6 in PPDC (μg).


(3) $$E=\frac{V_{E}\sum_{1}^{\mathrm{n–}1}C_{i}+V_{0}C_{n}}{m_{0}}$$

### Cell culture

The 4T1 cells were provided by Procell Life Science&Technology Co., Ltd (Wuhan, China) and cultured with RPMI-1640 containing 10% fetal bovine serum (FBS; SORFA, Beijing, China) and 1% penicillin/streptomycin in a 37°C incubator with 5% CO_2_.

The MCF-7 cells were provided by FuHeng Cell Center (Shanghai, China) and cultured with DMEM medium containing 10% FBS (Fujian Herui Biotechnology Co., Ltd) and 1% penicillin/streptomycin in a 37°C incubator with 5% CO_2_.

### *In vitro* cellular uptake

The 4T1 cells (1.0 × 10^5^ cells/ml, 0.2 ml) were seeded on confocal dishes. After 24 h, the cells were incubated with 100 μl PPDC (including 5 μg/ml DHA) for 1, 2, 4, 6 and 8 h, respectively. After fixed with 4% paraformaldehyde for 15 min, rinsed with PBS and stained with DAPI for another 5 min, the confocal laser scanning microscopy (CLSM, Leica SP5, Germany) was used to observe.

To further quantitatively study the cellular uptake of PPDC, 4T1 cells (5.0 × 10^5^ cells/ml, 0.2 ml) were seeded in 12-well plates. After 24 h, PPDC (including 5 μg/ml DHA) was introduced and cultured with the cells for 1, 2, 4, 6 and 8 h, respectively. Then, the cells were collected, rinsed with PBS and dispersed in 200 μl PBS for flow cytometry (FCM, FACSCanto (TM)II, Becton, Dickinson and Company, USA) detection.

### Exploration of endocytosis pathway

The endocytosis inhibition experiments were studied to further understand the internalization pathway of PPDC. Three endocytosis inhibitors, including CPZ (20 μg/ml), amiloride (10 μg/ml) and nystatin (20 μg/ml), were used to inhibit the clathrin-mediated, macropinocytosis-mediated and caveolae-mediated pathway, respectively. We used 4°C treatment to inhibit the energy-dependent pathway. The 4T1 cells (2 × 10^5^ cells/ml, 0.5 ml) were seeded in 12-well plates. After overnight incubation, the cells were pretreated with the three endocytosis inhibitors and at 4°C for 2 h. Afterward, the cells were incubated with PPDC (including 5 μg/ml DHA) for 5 h. In the end, the cells were harvested and analyzed through a flow cytometer (FACSCanto (TM) II, Becton, Dickinson and Company, USA).

### *In vitro* cytotoxicity studies

Cells (4T1 or MCF-7, 5.0 × 10^3^ cells/well) were seeded in 96-well plates. After overnight incubation, the cells were incubated with different concentrations of free DHA, free Ce6, PEG-PCL and PPDC for another 24 h. For groups requiring laser irradiation, the cells were firstly incubated for 4 h and then exposure to laser (0.5 W/cm^2^, 5 min). Afterward, the cell viability was evaluated through MTT assay and determined using the following formula:


(4) $$\text{Cell viability}\;\left(\%\right)=\frac{{\text{OD}}_{\text{sample}}-{\text{OD}}_{\text{blank}}}{{\text{OD}}_{\text{control}\;}-{\text{OD}}_{\text{blank}}}\times 100\%.$$

### Detection of cellular ROS generation

The level of intracellular ROS generation was investigated with DCFH-DA. DCFH hydrolyzed from DCFH-DA can generate DCF with fluorescence when oxidized by the intracellular ROS, so the level of intracellular ROS can be determined by measuring the fluorescence intensity of DCF [24, 25]. The 4T1 cells (5 × 10^4^ cells/ml, 0.2 ml) were seeded on confocal dishes. After overnight incubation, the cells were cultured with free DHA (1 μg/ml) and PPDC (including 1 μg/ml DHA) for 4 h. Then, after rinsed with PBS, the cells were incubated with DCFH-DA in accordance with the instructions of the ROS Assay Kit. Subsequently, the cells were exposed to the laser (0.5 W/cm^2^, 5 min). Finally, the cells were stained with Hoechst 33342 (20 min) for observation by CLSM.

### Cell apoptosis assays

The 4T1 cells (2 × 10^5^ cells/ml, 0.5 ml) were seeded in 12-well plates. After overnight incubation, the cells were treated with free DHA (1 μg/ml), free Ce6 (1 μg/ml) and PPDC (including 1 μg/ml DHA and 1 μg/ml Ce6) with/without laser irradiation, while the medium without drugs was set as the control group. For the irradiation groups, the cells were irradiated (0.5 W/cm^2^) for 5 min after 4 h of incubation. Cells in the different groups were all incubated for 24 h, then rinsed thrice, digested via trypsin and finally centrifuged to collect the cell precipitate. Eventually, the cells were stained in line with the instructions of the Annexin V-FITC Apoptosis Detection Kit and analyzed using flow cytometry.

## Western blot analysis

Western blot (WB) analysis was used to qualify protein biomarkers. The 4T1 cells were cultured until the cell density reached 70–80%. Then, the culture medium was replaced with fresh culture medium containing free DHA (1 μg/ml), free Ce6 (1 μg/ml) and PPDC (including 1 μg/ml DHA and 1 μg/ml Ce6), respectively, while the medium without drugs was set as the control group. After 4 h, the cells were irradiated (0.5 W/cm^2^) for 5 min and incubated for another 20 h. After that, the cells were collected and lysed with RIPA lysis buffer for 30 min. Then, the total proteins in cells were separated by centrifugation (14 000 rpm, 15 min, 4°C) and the protein concentration was measured using the BCA protein assay kit. After denaturation, the protein was separated by 12% sodium dodecyl sulfate–polyacrylamide gel electrophoresis (SDS-PAGE) and shifted to the polyvinylidene difluoride membranes. The membranes were blocked with western blocking buffer for 2 h at room temperature. Afterward, the membranes were hatched with primary antibody at 4°C overnight and hatched with secondary antibody at room temperature for 2 h. Finally, an ECL chemiluminescence kit and ChemiDoc imaging system (Bio Rad, USA) were used to observe the bands, and ImageJ software was used to analyze the gray values.

### *In vitro* hemolysis analysis

Fresh blood from healthy mice was collected into an anticoagulant tube. After centrifugation (1000 rpm) at 25°C for 10 min, red blood cells (RBCs) were collected and diluted to 2% (V/V) with PBS. Afterward, RBCs were incubated with PPDC (1.95, 3.91, 7.81, 15.62 and 31.25 μg/ml) at 37°C for 2 h and then centrifuged (12 000 rpm) at 25°C for 15 min to obtain the supernatant. Finally, the absorbance (545 nm) was detected using a microplate reader, and the hemolysis rate was calculated using the following formula through three parallel experiments. The PBS solution and 1% Triton X-100 solution were set as negative and positive control, respectively.


(5) $$\text{Hemolysis rate}\;(\%)=\frac{A_{\text{sample}}-A_{\text{negative}}}{A_{\text{positive}}-A_{\text{negative}}}\times 100\%.$$

### Animals and tumor model

Female BALB/C mice (6–8 weeks old, 18–22 g) were purchased from Shanghai Slac Laboratory Animal Co., Ltd. Each mouse was subcutaneously injected with 5 × 10^5^ 4T1 cells to establish the breast tumor model.

### Imaging studies

When the tumor size of nude mice reached 100–150 mm^3^, the nude mice were randomly divided into three groups (four mice per group). Mice were injected with saline (control), free Ce6 and PPDC at a dose of Ce6 2.5 mg/kg via tail vein. The fluorescence images were taken at predetermined time points (2, 4, 8, 12 and 24 h) by an IVIS Spectrum Imaging System (Perkin Elmer, MA, USA). Finally, the mice were sacrificed, and the main organs and tumors were dissected for *in vitro* fluorescence imaging. The fluorescence intensity was quantitatively analyzed through Living Image system software.

### *In vivo* anticancer efficacy

When the tumor size of mice reached approximately 100 mm^3^, the mice were randomly divided into seven groups (four mice per group). The groups were labeled as (A) saline (control), (B) saline+L (+L meant with Laser), (C) free DHA, (D) free Ce6, (E) free Ce6+L, (F) PPDC and (G) PPDC+L. The mice were treated every 2 days for four times. The i.v. injection doses of DHA and Ce6 were 5.0 and 5.7 mg/kg, respectively. After 24 h, the mice in B, E and G groups were irradiated (0.25 W/cm^2^, 5 min). The physical condition of the mice was monitored every day; meanwhile, the size of tumor and body weight of all mice were recorded every other day. The volumes of tumors were acquired through the formula below: Volume (*V*)  = (*a* × *b*^2^)/2 (*a* = length, *b* = width). The relative tumor volume was obtained with *V*/*V*_1_ (*V*_1_ is the initial tumor volume). On day 17, all mice were sacrificed and the tumors were dissected and weighed to evaluate the therapeutic effect of different treatment methods. The tumor growth inhibition (TGI) value was calculated based on the following expression: TGI (%) = (1 − mean tumor weight of treated group/mean tumor weight of control group)  × 100%.

## Results and discussion

### Characterization of nanoparticles

**Figure 1.**
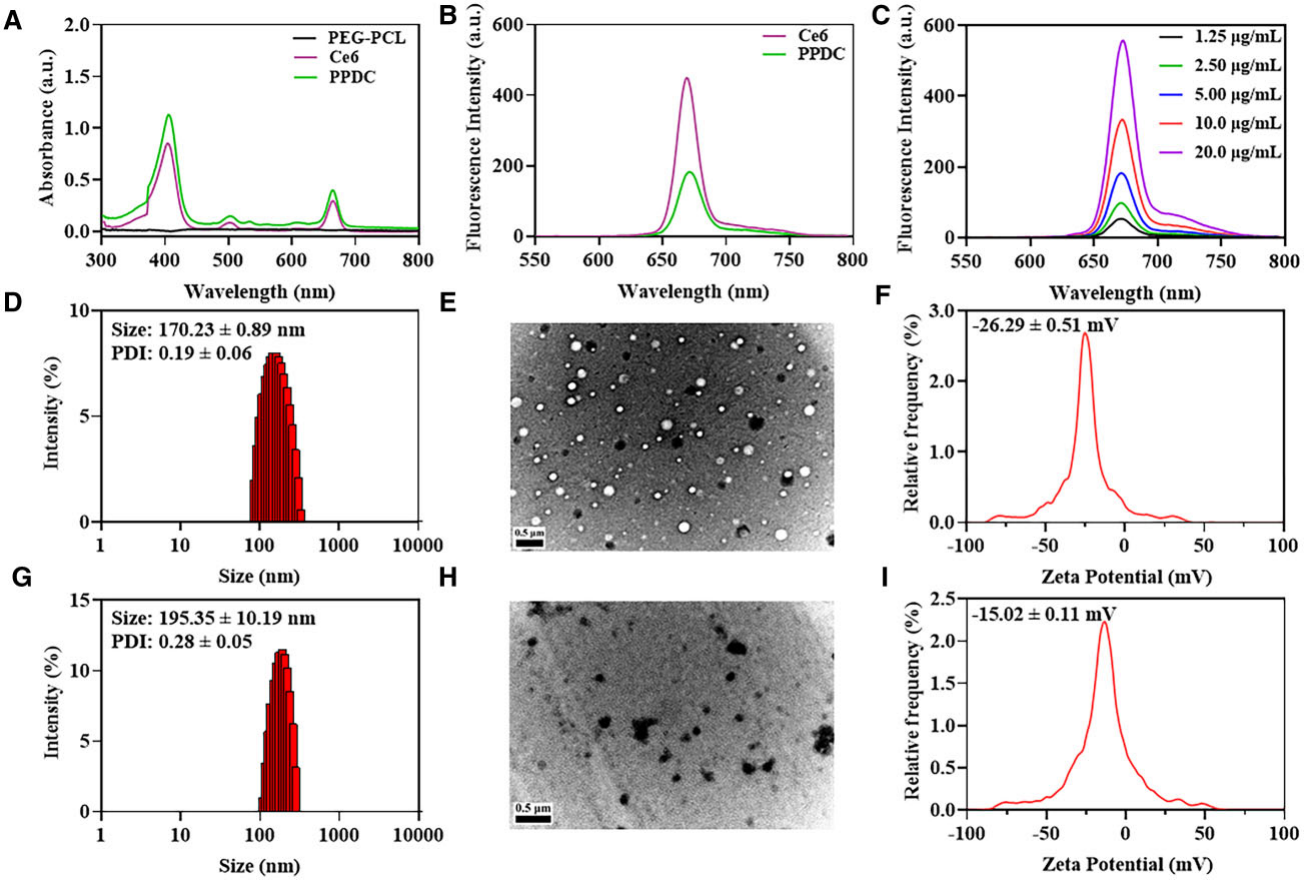
Characterization of DHA&Ce6@PEG-PCL (PPDC). (**A**) UV-vis absorption spectrum of PEG-PCL, Ce6 and PPDC. (**B**) Fluorescence emission spectra of Ce6 and PPDC. (**C**) Fluorescence emission spectra of PPDC with different concentrations. (**D**–**I**) Size distribution, TEM images and zeta potentials of PEG-PCL and PPDC. The scale bars of figure E and F were 0.5 μm.

To verify the successful preparation of PPDC, the ultraviolet absorption spectrum and fluorescence emission spectrum of different substances were compared (Fig. 1A). As shown in Fig. 1A–C and Supplementary Fig. S1A, all characteristic absorption peaks and fluorescence emission peaks corresponding to free DHA and free Ce6 were found in PPDC, and the fluorescence emission intensity increased gradually with the increase in PPDC concentration, indicating the successful encapsulation of DHA and Ce6. In addition, since the amphiphilic copolymer PEG-PCL could self-assemble into micelles in aqueous solution, we detected the critical micelle concentration (CMC) using a pyrene fluorescence probe method, which is 1.83 μg/ml (Supplementary Fig. S3). Then, we tested the content of DHA and Ce6 loaded in PPDC by ultraviolet absorption spectrophotometry. The LE of DHA and Ce6 was 17.8 ± 1.0% and 16.9 ± 0.8%, respectively, with the corresponding EE of 88.8 ± 4.9% and 84.5 ± 3.8% (Supplementary Table S1). As observed in this study, our prepared nanoparticles exhibited ideal drug loading and EE, which might be due to the hydrophobic parts (PCL) in the nanoparticles to preserve the hydrophobic drugs. In addition, the particle size and zeta potentials of PEG-PCL and PPDC nanoparticles were obtained through DLS analysis. The average particle size of PEG-PCL and PPDC nanoparticles was 170.23 ± 0.89 nm with poly-dispersity index (PDI) of 0.19 ± 0.06 (Fig. 1D) and 195.35 ± 10.19 nm with PDI of 0.28 ± 0.05 (Fig. 1G), respectively. It is worth noting that the hydrodynamic diameter of PPDC was larger than that of blank PEG-PCL (Fig. 1D), which might be due to the fact that drugs were loaded into the hydrophobic core of the nanoparticles, leading to the swelling of the nanoparticle core. Moreover, the morphology of PEG-PCL and PPDC nanoparticles was observed by TEM. As shown in Fig. 1E and H, PEG-PCL and PPDC nanoparticles were spherical particles with good dispersion, and the size was consistent with the particle size distribution profiles obtained from the DLS analysis. The zeta potentials of PEG-PCL (Fig. 1F) and PPDC (Fig. 1I) were −26.29 ± 0.51 and −15.02 ± 0.11 mV, respectively. The negative surface charge of PPDC was conducive to keeping nanoparticles stealthy and stable in blood circulation and might prolong the blood circulation time of drugs.

### Detection of ROS generation *in vitro*

**Figure 2.**
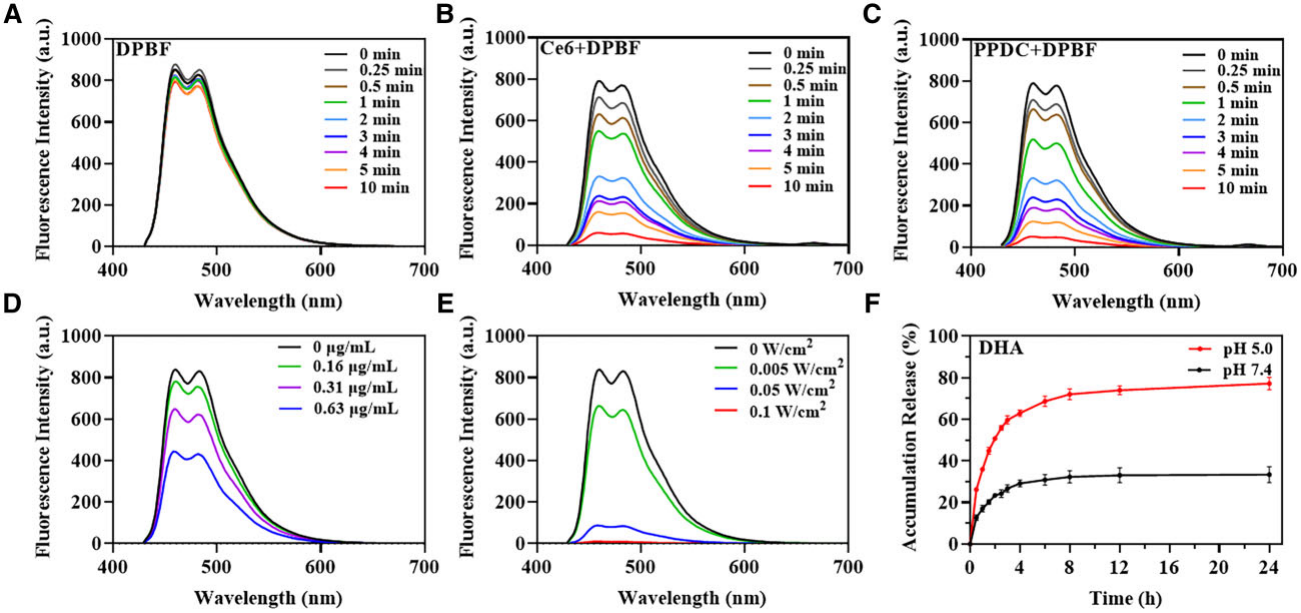
Detection of ROS with DPBF (10 μg/ml) as the probe and drug release. (**A**–**E**) Fluorescence emission spectra of DPBF with different treatment conditions. DPBF only (A), DPBF in free Ce6 (B) and DPBF in PPDC (C) solution (equivalent to 0.63 μg/ml Ce6) were upon laser irradiation for different durations (660 nm, 5 mW/cm^2^), respectively. (D) DPBF in PPDC solution with different concentrations (equivalent to Ce6) was irradiated by laser (660 nm, 3 min, 5 mW/cm^2^). (E) DPBF was irradiated with laser of different laser power in PPDC solution (equivalent to 0.31 μg/ml Ce6 concentration). (**F**) The cumulated release curve of DHA from PPDC in PBS (pH 7.4 or 5.0).

Since the generation of ^1^O_2_ is a crucial step in PDT, the ability of PPDC to generate ^1^O_2_ was assessed through the universal probe DPBF. As shown in Fig. 2B and C, the fluorescence intensity of DPBF with Ce6 or PPDC decreased continuously and significantly with the increase in laser irradiation time. By contrast, the fluorescence intensity of DPBF only was attenuated in a very small range (Fig. 2A), which might be caused by the inevitable photodegradation. The ultraviolet absorption of DPBF at 417 nm also showed a similar change with the same method (Supplementary Fig. S1B). Moreover, the effects of laser power and PPDC concentrations on ROS generation were further evaluated. The fluorescence intensity of DPBF decreased with the increase in PPDC concentration and laser power density shown in Fig. 2D and E. These results suggested that PPDC could generate ROS under laser irradiation, and the efficiency of ROS generation was found to be time-dependent, concentration-dependent and power-dependent. The above results implied that our prepared nanoparticles (PPDC) loaded Ce6 effectively, maintained a good ROS generation efficiency and could be a potential drug delivery system for antitumor therapy.

### Drug release assays

To investigate drug release, two types of buffers (pH 7.4 and 5.0) were used to mimic the normal physiological environment and tumor acidic microenvironment, respectively. It could be observed (Fig. 2F) that around 12.6% and 26.2% DHA were detected to release in pH 7.4 and 5.0 buffer at 0.5 h, respectively. After 4 h, the release amount of DHA in pH 7.4 and 5.0 buffer was 29.2% and 63.0%, respectively. After 24 h, more than 75% of DHA was released in pH 5.0 buffer, while only 33.4% of DHA was released in pH 7.4 buffer. The data manifested that PPDC was more sensitive in acidic environment, which was conducive to the selective release of drugs in tumor tissues. In addition, Ce6 was released more slowly, which could avoid rapid clearance of Ce6 *in vivo* (Supplementary Fig. S1C). The amount of released Ce6 at pH 7.4 was higher than that at pH 5.0, which might be due to the better hydrophilicity of Ce6 at high pH [26], which made it easier to leak out of the dialysis bag. DHA and Ce6 showed sustained release, and the release curve was in accordance with the two-phase drug release system, which was fast first and then slow. Hence, the fast diffusion of drugs from PEG-PCL polymer matrix likely led to the rapid release of the first phase, and the subsequent slow release of the second phase was mediated by the degradation of the polymer matrix [27]. These results showed that PPDC had the potential to achieve sustained drug release in tumor tissue.

### Cellular uptake

**Figure 3.**
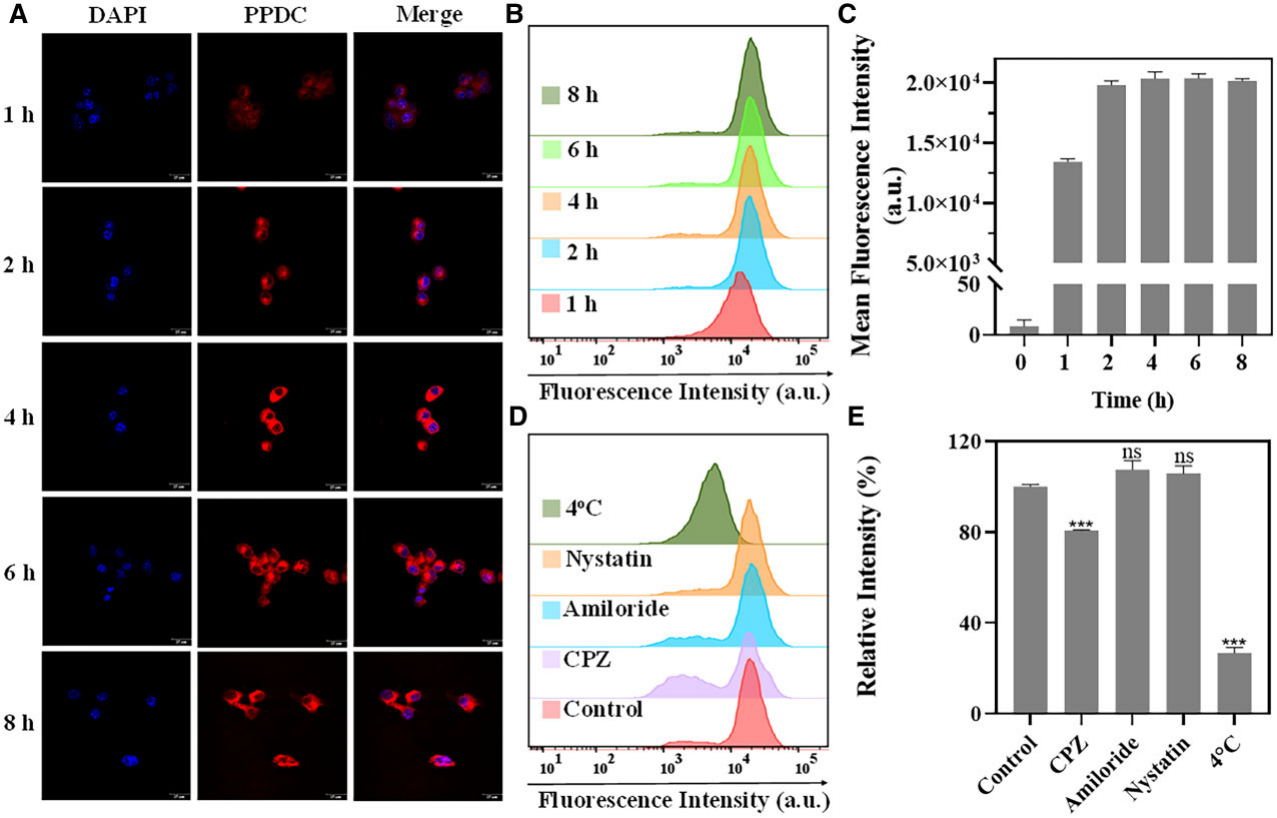
Cellular uptake and uptake mechanism study of PPDC. (**A**) CLSM images of 4T1 cells treated with PPDC for 1, 2, 4, 6 and 8 h. Scale bar: 25 μm. Blue fluorescence indicated the nucleus stained by DAPI (4ʹ, 6-diamidino-2-phenylindole) and red fluorescence originated from Ce6 in PPDC. (**B**, **C**) Flow cytometry profiles and quantitative analysis of cell uptake. (**D**, **E**) Flow cytometry profiles and quantitative analysis of endocytosis pathway. Chlorpromazine (CPZ, clathrin inhibitor), amiloride (macropinocytosis inhibitor) and nystatin (caveolin inhibitor) were used to pretreat the 4T1 cells, respectively. Results represent mean ± SD (*n* = 3). *** indicates *P *<* *0.001 and ns represents no significance versus control group.

To study the cellular internalization efficiency, cellular uptake of PPDC was first studied through CLSM. The images of CLSM suggested that compared with untreated cells, red fluorescence derived from PPDC loaded with Ce6 was obvious after incubation with PPDC for 1 h, and the brightest signal was found at 4 h (Fig. 3A). In addition, the quantitative detection results of nanoparticle internalization in 4T1 cells by FCM were consistent with confocal fluorescence imaging (Fig. 3B and C). The uptake reached a peak at 4 h and was 1.49-fold higher than that at 1 h. Taken together, the above results indicated that PPDC could be taken up by tumor cells at a fast rate, thereby promoting the accumulation of drugs in the cells, and was beneficial for the subsequent PDT.

### Exploration of endocytosis pathway

The endocytosis efficiency of the nanoparticles is closely related to the endocytosis pathway. To investigate the cellular uptake pathway of PPDC, 4T1 cells were treated with four inhibition methods of endocytosis pathway. Clathrin-mediated and macropinocytosis-mediated endocytosis pathways could be suppressed with CPZ and amiloride [28, 29], respectively. A key role is played by lipid raft in caveolae-mediated endocytosis pathway, and nystatin can deeply restrain the pathway through intercepting lipid raft [30]. In addition, 4°C treatment could cut off the energy supply of cells to inhibit energy-dependent pathway. As shown in Fig. 3D and E, compared with the control group, the cellular uptake of PPDC was reduced to 26.74% at 4°C, signifying that the internalization of PPDC was a process of energy consumption. After incubation with CPZ, the cellular uptake was reduced to 80.64%, while there was little difference after incubation with amiloride and nystatin. The above results showed that the cellular uptake of PPDC was mainly related to clathrin-mediated and energy-dependent pathway, which provided an important basis for the effective cellular uptake of PPDC.

### *In vitro* cytotoxicity studies

**Figure 4.**
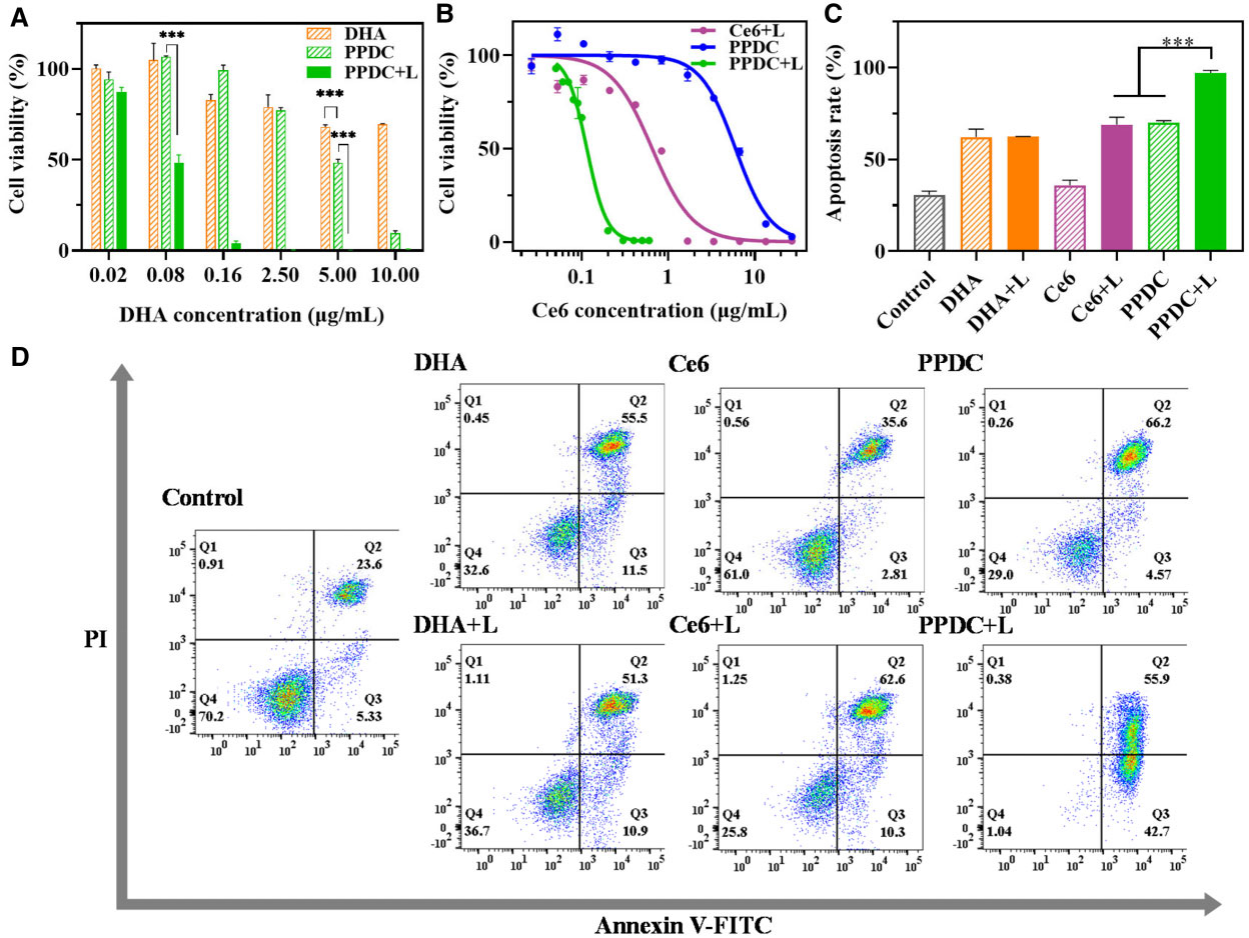
Cytotoxicity assays. (**A**, **B**) Cell viability was determined by MTT assay. The cell viability of 4T1 cells treated with free DHA, free Ce6 and PPDC for 24 h with or without laser irradiation. The curves were fitted by the GraphPad prism. (**C**) Quantification of the percentage of apoptosis in 4T1 cells. Results represent mean ± SD (*n* = 3). (**D**) The Annexin-V/PI apoptosis assay of 4T1 cells treated with free DHA, free Ce6 and PPDC for 24 h with or without laser irradiation was measured by flow cytometry. +L means that the cells of this group were irradiated (0.5 W/cm^2^) for 5 min. ^*^ and ^***^ indicate *P *<* *0.05 and *P *<* *0.001, respectively.

To study the therapeutic efficiency *in vitro* of free DHA, free Ce6 and PPDC with or without laser irradiation (660 nm, 0.5 W/cm^2^, 5 min), the cell viability was assessed by MTT assays. As shown in Fig. 4A and B, PPDC markedly strengthened the cytotoxicity of DHA (Fig. 4A) and Ce6 (Fig. 4B and Supplementary Fig. S2B), reducing their half-maximal inhibitory concentration (IC_50_) values on 4T1 cells from 9.50 to 3.38 μg/ml and from 0.65 to 0.10 μg/ml, respectively (Supplementary Tables S2 and S3). When the concentration of DHA was 5.00 μg/ml, the cell viability of the PPDC group was much lower than that of the free DHA group. While the cell viability ratio of the PPDC with laser irradiation (PPDC+L) group was close to 0% (Fig. 4A). Compared with the Ce6 with laser irradiation (Ce6+L) group, the PPDC+L group showed higher phototoxicity at low doses (Fig. 4B). In addition, the MCF-7 cell line was selected to further examine the effect of the combined therapy. As shown in Supplementary Fig. S2D, the PPDC+L group showed stronger lethality than other groups with 0.14 μg/ml DHA and 0.12 μg/ml Ce6. The above experimental results showed clearly that PPDC + L had an excellent effect in killing 4T1 and MCF-7 tumor cells, which was mainly attributed to the fact that PPDC could enter the cells with the superior cellular uptake efficiency, and the combination of chemotherapy and PDT greatly enhanced the inhibition of tumor growth. The combination therapy had potential application in the treatment of breast cancer.

Furthermore, considering the above research results, we discussed other potential factors affecting cell growth, including the toxicity of PEG-PCL and free Ce6 and the effect of laser irradiation on cell growth. As shown in Supplementary Fig. S2A–C, no significant cytotoxicity was observed in the groups of PEG-PCL and free Ce6 without laser irradiation, and the cells treated with free DHA groups (with or without laser irradiation) showed the similar survival level. These results indicated that PEG-PCL was of fine biocompatibility, and laser with a power density of 0.5 W/cm^2^ was safe for the cells. The above results proved that the toxicity of PPDC to cells was caused by drugs rather than by PEG-PCL.

### Cell apoptosis assays

Tumor cells can proliferate and transmigrate indefinitely partly due to the reduction or loss of their ability to undergo apoptosis [31]. Apoptosis is one of the most common mechanisms of cancer cell death, and one of the main mechanisms by which antitumor drugs inhibit tumor growth [32]. For this reason, we examined the effect of different materials on apoptosis. As shown in Fig. 4C and D, the cell apoptosis rates of the DHA group, PPDC group and Ce6+L group were 62.1%, 70.0% and 68.9%, respectively. The cell apoptosis rate of the PPDC+L group was the highest, reaching 97.1%, which was 1.6-fold, 1.4-fold and 1.4-fold higher than that of the DHA group, PPDC group and Ce6 + L group, respectively. The results suggested that the combined treatment of DHA and PDT could inhibit the proliferation of tumor cells by inducing apoptosis, and the apoptosis rate of tumor cells can be effectively improved by the combination therapy.

### Detection of cellular ROS generation

**Figure 5.**
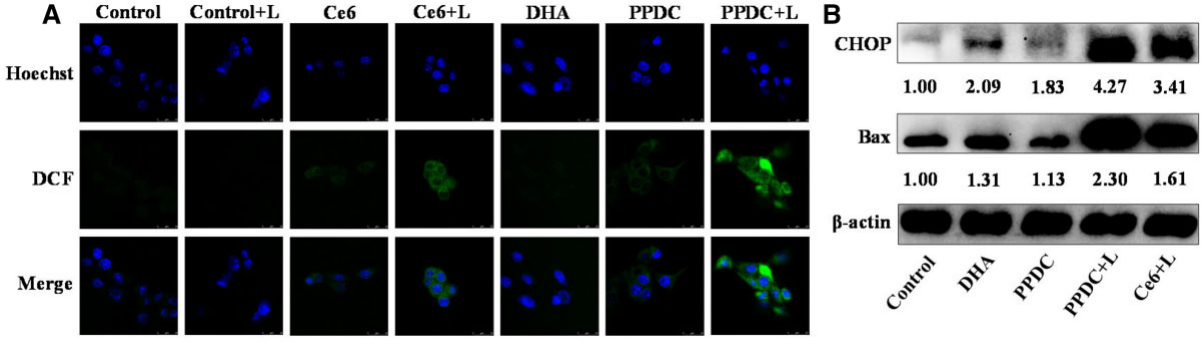
(**A**) Intracellular ROS generation of cells treated with free DHA, PPDC and PPDC with laser irradiation (0.5 W/cm^2^, 5 min). Blue fluorescence indicated the nucleus stained by Hoechst 33342 and green fluorescence was generated by ROS probe. Scale bar: 25 μm. (**B**) Western blot analysis of Bax and CHOP expression treated with free DHA, free Ce6 and PPDC in 4T1 cells for 24 h, and PBS as the control group.

Excessive ROS in cells affect the normal growth of the cells or kill the cells [33]. In this study, DCFH-DA fluorescence probe was used to explore ROS generation level in the cells. As shown in Fig. 5A, no obvious fluorescence was found in the control group with (+L) or without laser irradiation. Meanwhile, we did not observe clear fluorescence in the Ce6 group in the absence of laser. Moderate-intensity green fluorescence was found in the Ce6 + L group, suggesting that ROS was generated by Ce6 upon laser irradiation. Previous studies have shown that compared with normal cells, tumor cells with higher levels of oxidative stress are more vulnerable to ROS damage induced by exogenous factors [20]. Therefore, ROS generated by PDT is beneficial for selectively killing tumor cells. Meanwhile, weak fluorescence was found in the groups of DHA and PPDC. Studies have shown that the abnormal generation of ROS mediated by DHA leads to changes in various signaling pathways in cells, including ER stress [34], thereby promoting abnormal growth and death of tumor cells. The strongest fluorescence signal was observed in the PPDC + L group, which was higher than that in the other groups, suggesting that PPDC + L had the strongest ROS generation capability and could be interpreted through the augmented ROS generation capability in the synergistic DHA and PDT. These results suggested that the combination therapy could generate more ROS and had great potential to kill tumor cells efficiently.

### Induction of ER stress

The increased intercellular ROS could disturb the protein-folding ability of ER, resulting in the accumulation of misfolded and unfolded proteins, which leads to ER stress [35]. To respond to ER stress, tumor cells activate a series of stress–response signaling pathways—called the unfolded protein response (UPR)—while excessive or persistent UPR activates the downstream apoptotic factors to promote cell apoptosis [36, 37]. The transcription factor CHOP is one of the markers of ER stress, and its high expression plays a significant role in apoptosis induced by ER stress. It has been reported that apoptosis-related molecules such as proapoptotic members of the Bcl-2 family (e.g. Bax, Bak, Bim, Puma) are the targets of CHOP-induced apoptosis [38]. Therefore, we detected the expression of CHOP and Bax by WB analysis to determine whether DHA and PDT could induce ER stress and promote apoptosis (Fig. 5B). By analyzing the ratio of the gray values of the experimental group to that of the control group, we found that the expression of CHOP and Bax was slightly lower in the PPDC group than in the free DHA group. The phenomenon may be caused by the wrapping of the micelles, which could lead to the reduced release of DHA. Meanwhile, the expression of CHOP and Bax in the PPDC+L group was significantly higher compared with the other groups, which was because the PPDC loaded with DHA and Ce6 combined the functions of both. Overall, these results suggested that the combined treatment of DHA and PDT could enhance ER stress of tumor cells, and then induce cell apoptosis, which could become a good combination for cancer treatment.

### *In vitro* hemolysis analysis

Hemolysis assay is a critical pointer to evaluate the safety of nanoparticles *in vivo*. The interaction of PPDC and RBCs was evaluated. PBS was set as a negative control, while Triton X-100 was set as a positive control. As shown in Supplementary Fig. S1D, the supernatant of the positive control group was red due to the rupture of RBCs, while the PPDC and negative control group were colorless because their RBCs were intact. Meanwhile, the hemolysis rate of the PPDC group was below 5% (Supplementary Fig. S1E). These results suggested that PPDC nanoparticles might be biosafe and have the potential for treatment *in vivo*.

### Imaging studies

**Figure 6.**
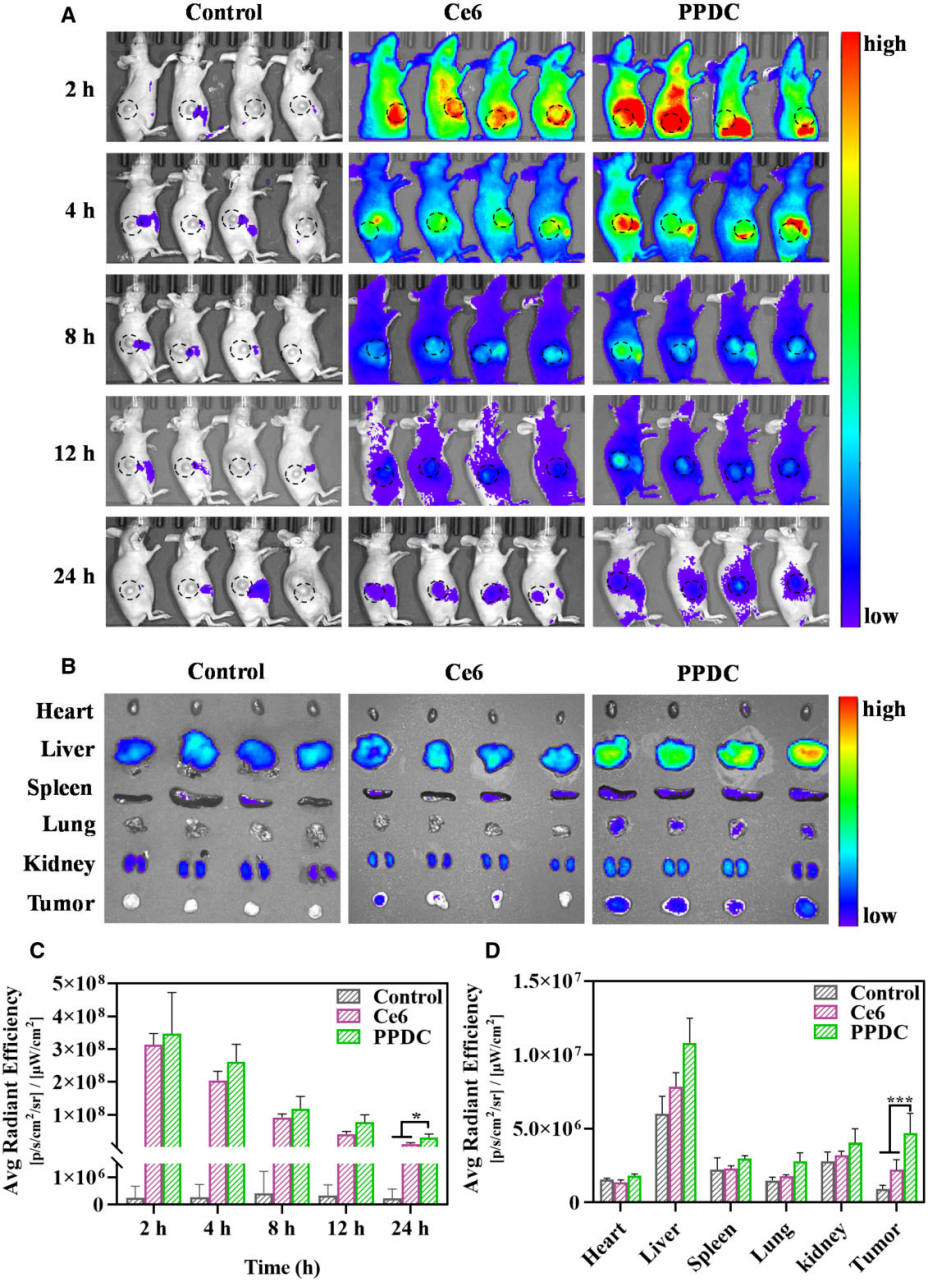
(**A**) *In vivo* fluorescence imaging of the mice bearing 4T1 tumor at preset time points (2, 4, 8, 12 and 24 h) after i.v. injection of free Ce6 and PPDC (the circle represented the tumor site). Saline was set as the control group. (**B**) *Ex vivo* fluorescence imaging of main organs (heart, liver, spleen, lung and kidney) and tumors dissected from the mice bearing 4T1 tumor 24 h after tail vein injection of free Ce6 and PPDC. (**C**) Semi-quantitative results of fluorescence intensity at tumor area *in vivo* fluorescence imaging (circled area in figure a). (**D**) Semi-quantitative results of average fluorescence intensity in major isolated organs and tumors 24 h after administration. Results represent mean ± SD (*n* = 4). ^*^ and ^***^ indicate *P *<* *0.05 and *P *<* *0.001, respectively.

The near-infrared fluorescence imaging method was applied to evaluate the tissue distribution of free Ce6 and PPDC *in vivo*. As shown in Fig. 6A, there was strong fluorescence in tumor tissues in the free Ce6 group and the PPDC group 2 h after administration. Notably, the fluorescence intensity of tumor tissues in the free Ce6 group was significantly weaker than that in the PPDC group after 12 h. The fluorescence quantification of tumor area (Fig. 6C) showed that the average fluorescence intensity of tumor tissues in the PPDC group was higher than that of the free Ce6 group at each time point. Moreover, tumors and organs were obtained for *ex vivo* fluorescence imaging at 24 h. As shown in Fig. 6B, the fluorescence of the tumor tissues in the PPDC group was brighter than that in the Ce6 group. Meanwhile, the fluorescence intensity of the tumors in the PPDC group was about 2.2-fold higher than that in the free Ce6 group (Fig. 6D). This indicated that the accumulation of PPDC in the tumor tissues at 24 h was still significantly higher than that of free Ce6, which was consistent with the *in vivo* fluorescence imaging. The above results confirmed the superior accumulation of PPDC nanoparticles at the tumor sites, which might be explained by the passive targeting of the nanoparticles (EPR effects) and the negative charge of the nanoparticles that allowed them to prolong the retention time of therapeutic drugs and prevented the drug from being rapidly excreted. In addition, the nanoparticles had fine biocompatibility and safety and could be used as a potential antitumor drug *in vivo*.

### *In vivo* anticancer efficacy

**Figure 7.**
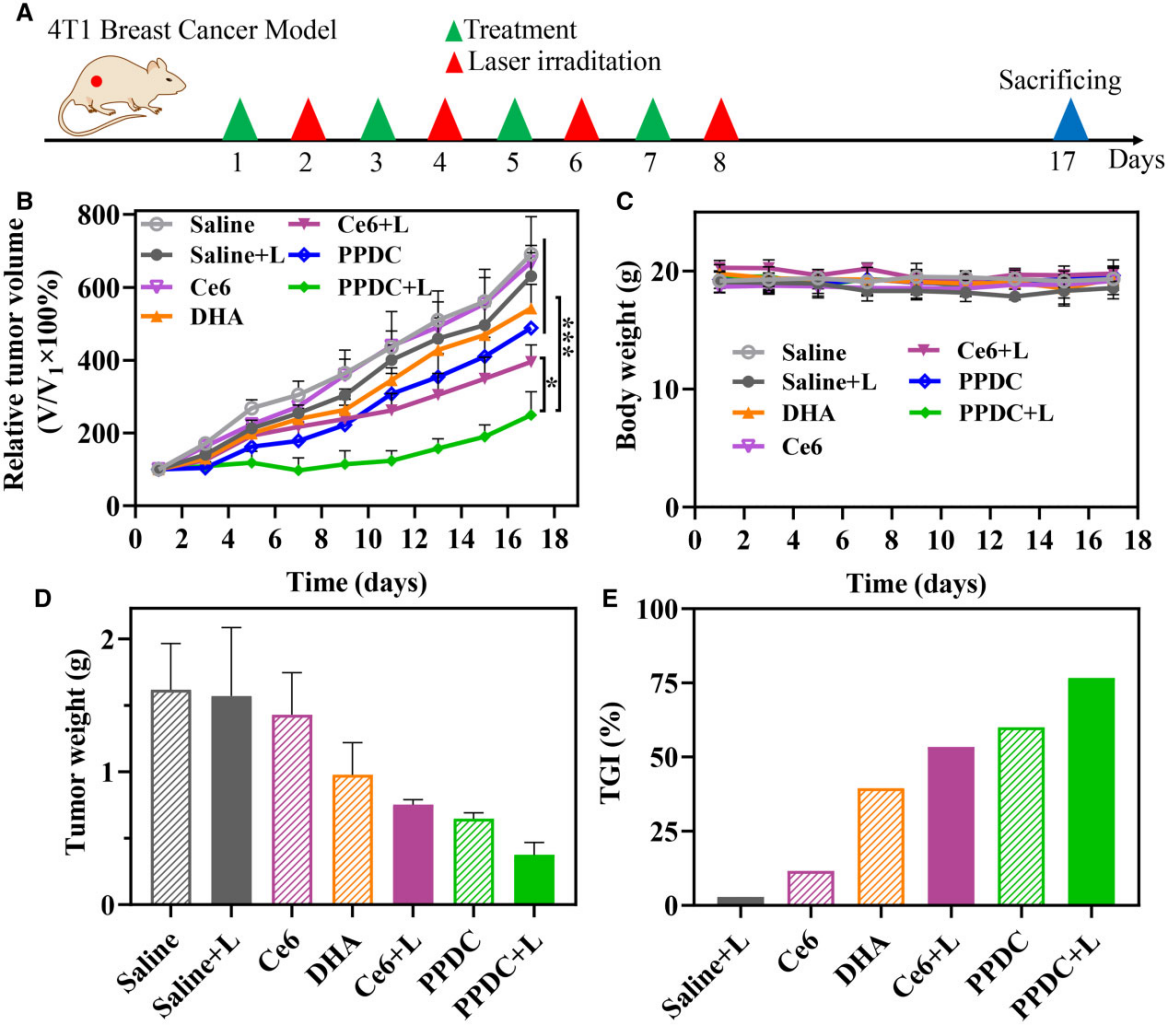
*In vivo* therapeutic effect of PPDC. (**A**) Experimental scheme. (**B**, **C**) The tumor growth curves (expressed by relative tumor volume) and body weight changes of 4T1 tumor-bearing mice in different treatment groups during the experiment period (+L means that the tumor of this group was irradiated with 660 nm laser at 0.25 W/cm^2^ for 5 min). (**D**) Weights of tumors harvested from the sacrificed mice on the 17th day. (**E**) Tumor growth inhibition rate (TGI) of different groups. Results represent mean ± SD (*n* = 4). ^*^ and ^***^ represent *P *<* *0.05 and *P *<* *0.001, respectively.

Subsequently, we further evaluated the antitumor activities of PPDC in mice bearing 4T1 orthotopic tumors. DHA, Ce6 and PPDC were i.v. administered every 2 days for a total of four times as scheduled (Fig. 7A). It is noteworthy that in considering the fine ROS generation performance of Ce6 and PPDC based on the previous research results, a low power density laser (0.25 W/cm^2^) was used on the premise of ensuring sufficient safety. After 17 days of treatment, the tumors were excised and weighed (Fig. 7D). As shown in Fig. 7B and D, there was no significantly inhibited growth behavior in the saline group, saline with laser group and Ce6 group in terms of tumor volume and tumor weight. By contrast, the PPDC + L group exhibited significantly higher antitumor activity than the other six groups. It is worth mentioning that the tumor thickness of the PPDC + L group was thinner than that of the other groups. In addition, the average tumor inhibition rate of the PPDC + L group (76.7%) was higher than those of the saline with laser (saline + L, 2.9%), Ce6 (11.6%), DHA (39.5%), Ce6 + L (53.4%) and PPDC (60.0%) groups (Fig. 7E), indicating the superior therapeutic efficacy of PPDC + L. Importantly, no significant change in body weight occurred in the mice receiving PPDC with or without laser (Fig. 7C), further suggesting that the combined application of chemotherapy and PDT through PPDC *in vivo* was safe. In conclusion, these data effectively confirmed the notable anticancer efficacy and the high biosafety of PPDC with low power density laser.

## Conclusion

In this study, we successfully constructed a PPDC with high drug loading and EE. The ROS generation experiment with DPBF as the probe demonstrated that PPDC had excellent ROS generation performance. By conducting a series of *in-vitro* and *in-vivo* experiments and researches within tumor, we showed that PPDC could be quickly taken up by cells to play the antitumor role of the drugs, produce a large amount of ROS with laser irradiation to trigger ER stress, and enhance cell apoptosis; the growth of tumors was further inhibited by combining it with Ce6-mediated PDT. The combination therapy combined the advantages of the two treatment methods as they complemented each other to inhibit tumor growth further. This work provided a potential application strategy for the treatment of breast cancer.

## Supplementary Material

rbad048_Supplementary_Data
