# Supplementary material for: Codelivery of dihydroartemisinin and chlorin e6 by copolymer nanoparticles enables boosting photodynamic therapy of breast cancer with low-power irradiation
Source: Regen Biomater. 2023 Apr 28;10:rbad048. doi: 10.1093/rb/rbad048 (PMC10224804; doi:10.1093/rb/rbad048)
Supplement: rbad048_Supplementary_Data [file rbad048_supplementary_data.docx]

Supplementary Information

Codelivery of Dihydroartemisinin and Chlorin e6 by Copolymer Nanoparticles Enables Boosting Photodynamic Therapy of Breast Cancer with Low-Power Irradiation

Jing Jia ^1,2,†^, Wenping Chen ^1,†^, Long Xu ^3^, Xuewen Wang ^1^, Min Li ^1^, Bin Wang ^1^, Xiangyu Huang ^1^, Tao Wang ^4^, Yang Chen ^5^, Mengdie Li ^1^, Dan Tian ^1^, Junyang Zhuang ^1,^* , Xinhua Lin ^2,^*, and Ning Li ^1,^*

^1^ Fujian Key Laboratory of Drug Target Discovery and Structural and Functional Research, School of Pharmacy, Fujian Medical University, Fuzhou 350122, China

^2^ Key Laboratory of Nanomedical Technology (Education Department of Fujian Province), School of Pharmacy, Nano Medical Technology Research Institute, Fujian Medical University, Fuzhou, Fujian 350122, China

^3^ School of Materials Science and Chemical Engineering, Ningbo University, Ningbo 315211, China

^4^ School and Hospital of Stomatology, Fujian Medical University, Fujian Stomatological Hospital, Fuzhou 350002, China

^5^ Department of Hepatobiliary Surgery, Fuzhou Second Hospital, Fuzhou 350007, China

^*^ Correspondence: ningli@fjmu.edu.cn or fjgtlny@126.com (N. L.); 13906939638@163.com(X. L.); Junyang.Zhuang@hotmail.com (J. Z.)

^†^ These authors contributed equally to this work

**Methods**

**Critical micelle concentration (CMC)**

The pyrene fluorescence probe method was used to measure CMC of polymeric micelles of PEG-PCL. In brief, the polymeric micelles were diluted to different concentrations with H_2_O, pyrene was dissolved to a concentration of 6×10^−5^ mol/L with acetone, then 10 μL pyrene was added into the polymeric micelles solution with different concentrations. After mixing, the solution was placed at room temperature to allow acetone to volatilize. Then the excitation spectra (emission wavelength: 395 nm) of solution was measured. The ratios of the fluorescence intensity at 337 nm to the fluorescence intensity at 334 nm were calculated and plotted against the decadic logarithm (log10) of concentration to determine the CMC value.

**Results**

Figure S1. (A) UV-vis absorption spectrum of DHA and PPDC. (B) The change of ultraviolet absorption intensity of DPBF in free Ce6 and PPDC solution (equivalent to Ce6 concentration of 0.63 μg/mL) with laser irradiation for different durations (660 nm, 5 mW/cm^2^) was detected by microplate reader, respectively. (C) The cumulated release curve of Ce6 from PPDC. (D) Photos of hemolysis test. (E) Hemolysis rate of red blood cells in PBS, Triton X-100, and different concentrations of PPDC.

Table S1. Drug loading efficiency (LE) and encapsulation efficiency (EE) of PPDC.

Table S2. The IC_50_ of DHA for 4T1 cells measured by MTT.

Table S3. The IC_50_ of Ce6 for 4T1 cells measured by MTT.

Figure S2. (A) The cell viability of 4T1 cells treated with PEG-PCL for 24 h. (B) The cell viability of 4T1 cells treated with free Ce6 for 24 h with or without laser. (C) The cell viability of 4T1cells treated with free DHA for 24 h with or without laser irradiation. (D) The cell viability of MCF-7 cells treated with free DHA, free Ce6 and PPDC for 24 h with or without laser irradiation. Laser: 660 nm, 0.5 W/cm^2^, 5 min. Results represent mean ± SD (n = 3). ^*^ and ^***^ indicate *p* < 0.05 and *p* < 0.001, respectively; ns represents no significance.

Figure S3. Critical micelle concentration (CMC) of polymeric micelles of PEG-PCL.
